# Urinary interleukin-18 as an early indicator to predict contrast-induced nephropathy in patients undergoing percutaneous coronary intervention

**DOI:** 10.3892/etm.2014.1898

**Published:** 2014-08-11

**Authors:** HAIYAN HE, WENHUA LI, WENHAO QIAN, XIN ZHAO, LIN WANG, YAREN YU, JIALI LIU, JING CHENG

**Affiliations:** Department of Cardiology, Affiliated Hospital of Xuzhou Medical College, Xuzhou, Jiangsu 221002, P.R. China

**Keywords:** interleukin-18, coronary artery angiography, contrast-induced nephropathy

## Abstract

Contrast-induced nephropathy (CIN) is at present the third leading cause of hospital-acquired acute kidney injury (AKI). Traditionally, it is diagnosed by measuring an increase of the serum creatinine (SCr) concentration. However, SCr is an insensitive marker for detecting CIN. This study was designed to investigate whether human urinary interleukin-18 (IL-18) is early predictive marker for CIN following coronary interventional procedures. The general clinical data of 180 patients who underwent coronary interventional procedures at the Department of Cardiology, Affiliated Hospital of Xuzhou Medical College from March 1, 2012 to September 31, 2012 were collected. A nonionic, low osmolality contrast agent was used in the laboratory at this time. SCr values and estimated glomerular filtration rate (eGFR) were measured prior to and within 24 and 48 h after the administration of contrast agents. Urine samples were collected prior to and 2, 6, 12, 24 and 48 h after the coronary interventional procedure, and urinary IL-18 levels were measured using an ELISA kit. CIN was defined as an increase of ≥0.5 mg/dl or ≥25% in SCr concentration over baseline 24–48 h after the procedure. CIN occurred in 16 of 180 (8.9%) patients. The levels of urinary IL-18 measured 2 h after the procedure were increased in the CIN group, but the increase was not significant (P>0.05). There were significant differences (P<0.05) between the urinary IL-18 levels 6, 12, 24 and 48 h after the procedure and those before the procedure. No significant difference was identified between the SCr levels measured prior to and 24 h after the procedure. The area under the receiver operating characteristic (ROC) curve of urinary IL-18 12 h after the procedure was 0.811 and the 95% confidence interval of the area under the curve was 0.735–0.888. If the critical point of the diagnosis of CIN was 815.61 pg/ml, the sensitivity was 87.5% and the specificity was 62.2%. Univariate analysis indicated that the urinary IL-18 level was positively correlated with the SCr concentration pre- and postprocedure. In conclusion, urinary IL-18 may be a promising indicator for the early prediction of CIN.

## Introduction

With an increasing number of patients receiving intravascular injections of iodinated contrast media every year worldwide, contrast-induced nephropathy (CIN) has become the third leading cause of hospital-acquired acute kidney injury (AKI) (1). CIN is a serious clinical problem associated with an increased morbidity and mortality rate, particularly in high-risk patients who have undergone coronary angiography and/or percutaneous coronary intervention. A vital step to lower the number of cases of CIN is to identify patients at risk and apply proven preventive interventions. A reduction in renal perfusion and toxic effects on the tubular cells caused by the direct and indirect effects of contrast media on the kidneys are generally recognized as important preventive mechanisms. Furthermore, contrast exposure causes a certain degree of imbalance between increased renal vasoconstruction and decreased vasodilatation. This leads to a decrease in renal blood flow and contraction of the afferent glomerular arteriole, as well as renal ischemia and cell necrosis (2). Oxygen radicals released by the ischemia-reperfusion not only contribute to renal damage but also the apoptosis of the renal tubular epithelial cells. In the majority of studies, the term CIN indicates an impairment in renal function, which is defined as an elevation in the levels of serum creatinine (SCr) following intravascular administration of the contrast media (3,4). However, SCr is a relatively inaccurate marker and alterations in the levels of SCr are not particularly sensitive or specific for small changes in the estimated glomerular filtration rate (eGFR). The levels of SCr may also be affected by a number of non-renal factors including age, ethnicity, muscle metabolism and nutrition.

A previous study has shown that the activation of glomerular mesangial cells causes the secretion of interleukin-18 (IL-18), affects the mitosis of the glomerular mesangial cells, promotes cell proliferation and induces the generation and releases a series of cytokines, which increases the accumulation of inflammatory cells within the glomerulus (5). Accumulating evidence indicates that urinary IL-18 is released in response to injury of the renal tubules (6,7), and may serve as an early biomarker of AKI. The present study was designed to investigate whether urinary IL-18 is an earlier predictive marker than SCr in CIN following coronary interventional procedures.

## Patients and methods

### Patient population

The general clinical data of 180 patients who underwent coronary interventional procedures at the Department of Cardiology, Affiliated Hospital of Xuzhou Medical College (Xuzhou, China) from March 1, 2012 to September 31, 2012 were collected in the study. Among them, 99 were male and 81 were female, with an average age of 66.5±9.1 years. Exclusion criteria were: i) the use of drugs with renal toxicity during the preoperative period; ii) severe hepatic and renal dysfunction, with severe renal dysfunction defined as eGFR <30 ml/min/1.73 m^2^; iii) tumors; iv) New York Heart Association class IV congestive heart failure or left ventricular ejection fraction (LVEF) <35%; v) thyroid or adrenal dysfunction; vi) acute or chronic infectious diseases; and vii) pregnant or breast-feeding females. A nonionic, low osmolality contrast agent was used in this test. The osmotic concentration was 800 mOsm/kg. All patients were routinely offered antiplatelet, anticoagulation, antiangina and conventional hydration treatment, as well as monitoring of lipids, blood pressure and blood glucose.

The study protocol was in accordance with the Declaration of Helsinki and was approved by Ethics Committee of the Affiliated Hospital of Xuzhou Medical College (Xuzhou, China). All patients provided written informed consent for the procedure.

### Laboratory assay

SCr and other biochemical indicators were measured. Fasting blood specimens were collected prior to and at 24 and 48 h after the procedure in the biochemical laboratory and subjected to analysis using an Olympus AU2700 Automatic Biochemistry Analyzer (Olympus, Center Valley, PA, USA) for determination. Urine specimens were collected prior to and at 2, 6, 12, 24 and 48 h after the procedure and immediately centrifuged (1,409 × g, 20 min, 4°C). The supernatant fractions were collected and stored frozen at −80°C until use. Urinary levels of IL-18 were measured using an ELISA kit purchased from R&D Systems (Minneapolis, MN, USA). All procedures were performed strictly following the manufacturer’s instructions. Renal function was assessed by the eGFR using the Modification of Diet in Renal Disease formula for Chinese patients (8): GFR (ml/min/1.73 m^2^) = 175 × SCr (mg/dl)^−1.1549^ × age^−0.2039^ (x 0.79 if female). This equation gives a more accurate assessment of renal function than SCr alone. CIN was defined as an increase of ≥0.5 mg/dl or ≥25% in SCr concentration over baseline 24–48 h after the intravascular administration of contrast medium, without an alternative etiology (9).

### Statistical analysis

Statistical analysis was performed by a professional statistics researcher. Continuous variables are expressed as the mean ± standard deviation. Student’s t-test and one-way analysis of variance were used for the comparison of continuous variables. Categorical data were presented as absolute values and percentages. The χ^2^ or the Fisher exact tests were used for the comparison of categorical variables. Pearson’s correlation analysis was used to evaluate correlations. All hypothesis testing was two-tailed. P<0.05 was considered as statistically significant. The SPSS version 16.0 (SPSS, Inc., Chicago, IL, USA) package was used for all calculations.

## Results

### Differences in SCr and eGFR values prior and subsequent to the procedure

These findings are shown in Table I. A total of 180 patients undergoing coronary interventional procedure were selected. CIN occurred in 16 of 180 (8.9%) patients. The 180 patients were divided into a CIN group (16 patients) and a non-CIN group (164 patients). No significant differences were identified in age, gender, body mass index, hemoglobin, SCr, eGFR and incidence of hypertension between the CIN and non-CIN groups. The medications and hydration volumes used during hospitalization were also not statistically different. The SCr peak level in the CIN group (105.94±38.29 μmol/l) was significantly higher than that in the non-CIN group (67.13±16.32 μmol/l) at 48 h after the procedure (P<0.05). There were significant differences (P<0.05) in the eGFR level at 24 and 48 h after the procedure between the CIN and non-CIN groups.

### Differences in urinary IL-18 level prior and subsequent to the procedure

These findings are shown in Table II. The urinary IL-18 levels were elevated 2 h after the procedure in the non-CIN and CIN groups, but no statistically significant difference was identified (P>0.05). The urinary IL-18 levels in the CIN group increased significantly at 6 and 12 h after the procedure compared with those in the non-CIN group (P<0.01), and began to decline 24 h after the procedure but remained higher than those in the non-CIN group (P<0.01). The IL-18 levels had not returned to the normal level 48 h after the procedure.

### Receiver operating characteristic (ROC) curve analysis

**Figure 1:**
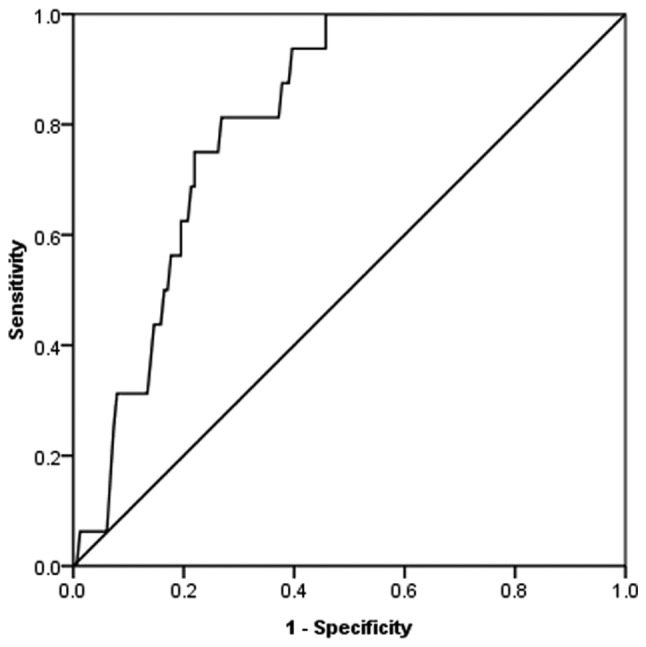
ROC analysis shows a high area under the curve for the 12 h postoperative urinary IL-18. At a cut-off level of >815.61 pg/ml, urinary IL-18 exhibited 87.5% sensitivity and 62.2% specificity for detecting contrast-induced nephropathy. Area under the ROC curve, 0.811; 95% confidence interval, 0.735–0.888; P<0.05. ROC, receiver operating characteristic; IL-18, interleukin-18.

The ROC curve for urinary IL-18 measured 12 h after the procedure is shown in Fig. 1. The area under the ROC curve of urinary IL-18 12 h after the procedure was 0.811, and the 95% confidence interval of the area under the curve was 0.735–0.888. If the critical point of the diagnosis of CIN was 815.61 pg/ml, the sensitivity was 87.5% and the specificity was 62.2% (P<0.05).

### Correlation between urinary IL-18 concentration and SCr

Bivariate analysis shows that the level of IL-18 prior to and within 24–48 h after the procedure positively correlated with SCr at the same time points. Preoperative urinary IL-18 and preoperative SCr (*r*=0.177, P=0.017), postprocedure 24 h urine IL-18 and postoperative 24 h SCr (*r*=0.174, P=0.019) and postprocedure 48 h urinary IL-18 and postoperative 48 h SCr (*r*=0.251, P=0.001) showed positive correlations.

## Discussion

With the increasing number of diagnostic and therapeutic catheterizations each year, particularly among patients who may have serious conditions predisposing them to CIN, for example old age, diabetes, renal insufficiency, cardiac insufficiency and the application of large doses of contrast medium, the incidence of CIN is likely to continuously increase. The ability to effectively prevent CIN in high-risk patients should provide significant public health benefits by potentially reducing the in-hospital mortality rate, the length of hospital stay and the subsequent use of chronic hemodialysis. SCr, although used routinely in clinical practice and in clinical trials, is currently considered a poor marker of acute renal dysfunction (6). Only when the GFR decreases to <50% of normal is SCr likely to rise. The detection of acute renal failure (ARF), which is characterized by a rapid decline in GFR, is based on an increase in SCr. However, there are limitations in the application of Cr for evaluating GFR. SCr cannot accurately reflect GFR in the unsteady state of ARF. Thus, minor changes in Cr, as observed in early ARF, may reflect a substantial decline in GFR. Therefore, the early diagnosis of CIN is crucial for prevention (10).

It is generally accepted that the main mechanism of CIN, with regard to contrast agent-induced hemodynamic changes and their effect on renal tubular epithelial cells, is by a direct toxic effect, with the massive production of free radicals intensifying the kidney function damage. Urinary IL-18 is an inflammatory factor expressed in the proximal convoluted tubule. It comprises 157 amino acid residues and has a molecular weight of ~18.3 kDa. It is activated by caspase-1 in the proximal convoluted tubule, breaks away from the cell, and is detected in urine (11). Urinary IL-18 has been shown to be specific for the diagnosis of ischemic renal injury (11). A prospective study involving 138 patients with AKI in an intensive care unit (ICU) showed that urinary IL-18 was a reliable test for the early diagnosis of AKI in critically ill patients (6). The pathogenesis of CIN remains controversial; however, but the main hypothesis is that it involves renal tubular local ischemia-reperfusion injury (12). Currently the only effective prevention measure for CIN is hydration therapy (13,14). Previously, the use of urinary IL-18 as biomarker of AKI has been investigated in patients in ICUs and in patients following cardiac surgery and kidney transplantation (6,7,15). All these studies using urinary IL-18 showed that the biomarker was able to diagnose AKI much earlier than SCr. The present study verified that urinary IL-18 could act as an improved early diagnostic marker for CIN.

The urinary IL-18 concentration and SCr levels were shown by the correlation analysis to have positive correlations pre- and postprocedure in the present study. In the CIN group, the concentration of SCr was statistically significantly different compared with the preoperative value 48 h after the procedure, the eGFR was statistically significantly different at 24 h after the procedure, while the urinary IL-18 level differed significantly 6 h after the procedure. Studies have also shown that the urinary IL-18 is at its peak 12–24 h after the administration of contrast agents (7,16). It may be concluded that the urinary IL-18 level increases earlier than SCr in CIN. Ling *et al* (16) examined 150 patients undergoing a coronary interventional procedure; the urinary IL-18 level increased significantly following the procedure, whereas SCr exhibited no evident change, compared with that in the non-CIN group. The study observed that postprocedure urinary IL-18 levels were clearly increased in the CIN group, which is consistent with the results of the present study. A study by Bulent *et al* (17) compared 15 cases of CIN with 36 control patients with regard to preoperative and 72 h postoperative urinary IL-18 concentration, and found no significant difference in urinary IL-18 concentration in the two groups of patients. The urinary IL-18 level in kidney injury following surgery reached a peak 12–24 h postoperatively, and returned to the baseline level 72 h postoperatively.

Urinary IL-18 can be measured using specific ELISAs, which are fast, reliable and low-cost, to test urine specimens from patients. This is a convenient and non-invasive technique, which therefore can be extended clinically. Postoperative urinary IL-18 is readily monitored, provides an early prediction of the occurrence of CIN, and enables proactive intervention measures to be taken to benefit patients. Therefore, the urinary IL-18 level is an improved indicator for the early prediction of CIN.

The limitations of this study are that the sample size was small and there were a variety of confounding factors. Further studies with larger sample sizes and increased monitoring of serum IL-18 and urine Cr concentrations over the same period, and the identification and exclusion of certain potential factors that may also cause increases in IL-18 levels, such as acute coronary syndrome, are required to validate the findings of the present study.

## Figures and Tables

**Table I:** Differences in the SCr and eGFR values prior and subsequent to the procedure in the two groups.

Groups	SCr (μmol/l)	eGFR (ml/min/1.73 m^2^)
Non-CIN		
Pre-procedure	70.04±19.25	95.30±22.56
Post-procedure		
24 h	65.92±15.81	106.10±23.61
48 h	67.13±16.32	98.65±23.23
CIN		
Pre-procedure	73.06±23.62	94.30±31.83
Post-procedure		
24 h	84.04±30.52	76.84±23.70^a^
48 h	105.94±38.29^a^	64.66±28.40^a^

a P<0.05 compared with the non-CIN group.

Data are presented as the mean ± standard deviation. SCr, serum creatinine; eGFR, estimated glomerular filtration rate; CIN, contrast-induced nephropathy.

**Table II:** Differences in the urinary IL-18 level (pg/ml) prior and subsequent to the procedure in the two groups.

		Post-procedure				
		
Groups	Pre-procedure	2 h	6 h	12 h	24 h	48 h
Non-CIN	590.80±298.66	661.60±297.50	782.89±270.52^a^	834.82±321.81^a^	773.99±264.69^a^	664.59±232.73
CIN	646.62±243.59	883.79±181.09	998.47±302.43^b^,^c^	1172.13±323.68^b^,^c^	1031.56±369.31^b^,^c^	843.92±284.11^d^

a P<0.01 and

b P<0.05 compared with pre-procedure;

c P<0.01 and

d P<0.05 compared with the non-CIN group.

CIN, contrast-induced nephropathy; IL-18, interleukin-18.
